# Evaluating the effectiveness of the Jennings disaster management model on nursing students’ knowledge and self-efficacy

**DOI:** 10.1186/s12912-025-04098-2

**Published:** 2025-12-11

**Authors:** Samar Mahmoud Ramadan, Magda Mohamed, Gehad Abo –Elmaty

**Affiliations:** 1 Department of Family and Community Health Nursing, Faculty of Nursing, Port Said University, Port Said City, Egypt; 2 Department of Clinical Research, Damietta Directorate of Health Affairs, Ministry of Health and Population, Damietta City, Egypt

**Keywords:** Disaster preparedness, Disaster management education, Nursing students, Self-Efficacy, Jennings disaster model, Emergency nursing

## Abstract

**Background:**

Disasters have a direct impact on the community, and nurses are the first health professionals to interact with people affected by disasters. So, the most common challenge faced by nurses is inadequate levels of preparedness, and equipping nursing students with disaster response skills is crucial. This study was conducted to evaluate the effectiveness of the Jennings disaster management model on nursing students’ knowledge and self-efficacy..

**Method:**

This study used a convenience sample method and a quasi-experimental one-group pre-post-test study research design to conduct it on 648 nursing students at Port Said University, Egypt in 2024. The Jennings Disaster Management Model (JDMM), a structured four-phase approach to disaster preparedness, guided the intervention. Two tools were used to collect data: the Disaster Management Knowledge Questionnaire and the Disaster Response Self-Efficacy Scale..

**Results:**

Post-intervention, the proportion of students with satisfactory disaster management knowledge rose from 0.8% to 75.0%, reflecting a significant improvement (*p* < 0.001). Mean self-efficacy scores also increased from 60.88 (SD = 15.12) to 83.27 (SD = 13.59), (*p* < 0.001).

**Conclusion:**

Integrating structured disaster management education into nursing curricula substantially improves students’ preparedness, fostering individual competence and enhancing community resilience. Future research should examine long-term knowledge retention and practical application in real-world settings..

**Supplementary Information:**

The online version contains supplementary material available at 10.1186/s12912-025-04098-2.

## Introduction

A disaster occurs when society is severely disrupted, and a large number of individuals are impacted. Disaster preparedness and nursing abilities are vital and beneficial, and nurses play a vital role in disaster response. Disasters, whether natural or man-made, disrupt societies, overwhelm local capacities, and necessitate external aid [1]. Their frequency and intensity are escalating due to climate change, population growth, and geopolitical instability, challenging global healthcare systems [2]. Effective disaster preparedness requires knowledge of incident command, triage, and assessment protocols, with self-efficacy playing a crucial role in healthcare providers’ readiness [3, 4].

Exposing nurses to potential harm, nursing education can help individuals and communities impacted by disasters. Moreover, Nurses, as frontline responders, need specialized training to enhance their disaster management competencies [5]. The Jennings Disaster Nursing Management Model provides a structured approach through four phases-pre-disaster, disaster, post-disaster, and outcome optimization [6]. The International Council of Nurses (ICN) highlights eight core competencies essential for disaster response, underscoring the importance of integrating disaster training into nursing education to strengthen healthcare resilience [7–9].

Historically, Egypt witnessed several types of disastrous events that hit the country till nowadays. facing climate-related disasters, migration crises, and economic instability [10]. The spread of pandemic diseases such as COVID-19 makes horror worldwide and is considered a big disaster in the world. Despite the necessity, existing training programs often overlook self-efficacy and willingness to respond to disaster [3].

The Jennings Disaster Management Model serves as a powerful framework for enhancing nursing students’ self-efficacy in disaster preparedness. Self-efficacy-the belief in one’s ability to perform specific tasks effectively- is fundamental in enabling nursing students to act decisively during crises. This model offers a well-structured and integrative approach, targeting core components of disaster readiness including knowledge acquisition, strategic planning, rapid response, and recovery protocols. Its design is tailored to healthcare and nursing contexts, making it exceptionally aligned with the educational and practical needs of future nurses. By instilling confidence and preparedness, the Jennings Model contributes significantly to building a resilient and responsive healthcare workforce [11].

Since nurses make up the largest group of healthcare professionals, they are crucial to disaster preparedness efforts. Their responsibilities include risk identification, analysis, planning, drills, education, and training, as well as pinpointing areas that require improvement and development. To satisfy the demands of the respective serving community and to serve in a disaster, nurses should be prepared with the requisite skills and knowledge [12].

Finally, continuous structured disaster education is vital for improving mass casualty management and societal resilience [13]. Similarly, in order to effectively respond to a critical event or disaster, nurses must be able to respond, communicate, and create a new plan in the event that circumstances change unexpectedly. In addition, nurses must be involved in the creation of a disaster plan in order to be able to initiate one, and proper plan execution necessitates ongoing assessment, education, training, and drills [14]. Therefore, given these considerations, this study aims to evaluate the effect of applying the Jennings Disaster Management Model on the knowledge and self-efficacy response among nursing faculty students.

## Methods

### Study design and settings

This interventional study had a quasi-experimental one-group pre-post-test study conducted at the Faculty of Nursing, Port Said University, Egypt, in 2024.

### Population and sampling

This study included all nursing students from Port Said University’s four academic levels (648) —159 first year, 103 s year, 202 third year, and 184 fourth year. Participation was voluntary, and informed consent was obtained electronically. No exclusion criteria were applied.

### Sampling strategy

A convenience sampling method was employed due to practical considerations beyond logistical constraints, including political instability, which frequently disrupts academic schedules, making probability sampling difficult [15]; and ensuring a high response rate (95.8%), capturing a broad representation of nursing students across different academic years.

### Data collection and instrument

In this study, after obtaining ethical approval and necessary permissions. Students were recruited via structured WhatsApp groups corresponding to their academic levels. Informed consent was obtained electronically. Participants completed anonymous, self-reported online questionnaires two weeks after the intervention. To comprehensively assess nursing students’ disaster management knowledge and self-efficacy in disaster response, the study employed two validated tools:

**The Disaster Management Knowledge Questionnaire (DMKQ)**, adapted from Arun Jothi (2015) [16]. assesses students’ disaster knowledge across and includes 47 items and five domains: personal characteristics (7 items), disaster concepts (11 MCQs), disaster types (3 MCQs), disaster management strategies (19 MCQs), and preventive/rehabilitation measures (7 MCQs). A binary scoring system (1 = correct, 0 = incorrect) classified knowledge as satisfactory (≥ 76%), average (51%–75%), or unsatisfactory (≤ 50%). Validity was confirmed by seven experts, and internal consistency was high (Cronbach’s α = 0.90).

**The Disaster Response Self-Efficacy Scale (DRSES)**, adapted from Cruz et al. (2022) [17], measures confidence across three domains: rescue on-site, disaster psychological nursing, and role adaptation. The 19-item, 5-point Likert scale (1 = no confidence to 5 = complete confidence) classified confidence as positive (>3), basic (= 3), or negative (< 3). Validity was confirmed by seven experts, and reliability was excellent (Cronbach’s α = 0.939). A pilot study was conducted on 10% of the total sample (62 students) to test the feasibility, applicability, and understandability of the instruments. The subjects of the pilot study were not included in the actual study sample.

### Educational intervention

Data collection took a period of six months, beginning on 1st January and ending in June 2024, and the study was divided into four phases: assessment, planning, intervention, and evaluation.

**Phase I: Assessment (Pre-Test Phase)** before implementing the educational intervention, a baseline assessment was conducted to evaluate students’ knowledge and confidence in disaster management. Although fourth-year students may receive limited exposure to disaster-related topics through individual lectures within certain courses, there is no structured or comprehensive disaster management curriculum implemented across any academic year at Port Said University. Assessment utilized two standardized tools: the Disaster Management Knowledge Questionnaire (DMKQ) and the Disaster Response Self-Efficacy Scale (DRSES). The researcher spent six months conducting fieldwork, interviewing each student individually for approximately 30–35 min using the previously described research methods. Additionally, the data was obtained during these phase sessions (pre-test), and the confidentiality of all gathered information was strictly protected.

**Phase II: Planning** during this phase, the researcher developed an instructional intervention based on relevant research findings and the specific needs identified during the nursing student assessment phase. This ensured that the intervention was tailored to address gaps in students’ knowledge and confidence in disaster management. As part of the intervention, the researcher created a comprehensive booklet, designed to enhance students’ understanding and disaster response self-efficacy. The content of the booklet was developed based on international disaster preparedness guidelines (including WHO and CDC), and current academic literature in nursing and public health. The selected content focused on the roles of nurses in disaster preparedness and response, ensuring relevance and clarity for undergraduate nursing students. Before distribution, the booklet was reviewed by a panel of three academic experts in disaster management and nursing education. Their feedback was incorporated to enhance the content’s validity and educational value. In addition, a pilot study was conducted on 10% of the study sample (62 students) to assess the clarity, language simplicity, and applicability of the tools, including the educational booklet. The pilot feedback was used to refine the booklet before final implementation. Participants in the pilot were randomly selected and excluded from the main study. The final version of the booklet was carefully assessed and validated to ensure accuracy and effectiveness. Its primary objective was to reinforce learning and improve students’ ability to respond to disasters effectively.

**Phase III: Implementation** The educational sessions were carried out at the Faculty of Nursing, Port Said University, with students divided into small groups (12–15 participants). Each group attended five structured training sessions. The researcher herself facilitated all the sessions, visiting the faculty, four times weekly. The implementation of the educational program was conducted as each group obtained three sessions a week, each session took an average of about an hour. To enhance student engagement and motivation, a variety of interactive teaching techniques were applied. These included the use of visual aids (e.g., posters and instructional videos), printed educational materials (e.g., booklets), and interactive discussions that encouraged student participation. Each session began with a review of the previous session’s content and a clear outline of the objectives for the current session, presented in simple and accessible language.

Motivational strategies were based on constructivist learning theory, which emphasizes active student involvement in constructing knowledge. Examples included asking open-ended questions, relating content to real-life disaster situations, and providing positive feedback to encourage participation. The researcher also responded to student inquiries in a supportive manner, fostering a safe and engaging learning environment.

#### Phase IV

Evaluation phase (Post-test) after the implementation of an educational program, post-tests were done immediately to evaluate the effect of the educational program on the difficulties and challenges of nursing students using the same tools in the pre-test with a comparative analysis of pre-and post-intervention scores quantifying program effectiveness.

### Statistical analysis

Data was analyzed using IBM SPSS Statistics v.23.0 (IBM Corp., Armonk, NY, USA). Categorical variables were summarized as frequencies and percentages, while continuous variables were described using mean, standard deviation, median, and range. Normality was assessed using the Kolmogorov-Smirnov test (*p* > 0.05 indicating normal distribution). For group comparisons, the Chi-square test was applied to categorical data, while McNemar and Marginal Homogeneity Tests were used to assess within-subject changes. Paired t-tests were employed for normally distributed continuous variables. Pearson’s correlation coefficient evaluated relationships between continuous variables. The significance level of *p* < 0.05 was considered statistically significant.

## Results

### Demographic characteristics of nursing students

Table 1 presents the demographic profile of the participating nursing students. The mean age was 20.48 ± 1.62 years, and (53.4%) were over 20 years old. Males comprised 52.5% of the sample, and 31.2% were in their third academic year. Notably, 90.0% of students had never received prior disaster training, while only 10.0% had attended at least one disaster training course. Among those who participated, 35.4% had completed a three-day training program.

**Table 1:** Distribution of the nursing students studied according to their personal characteristics (N = 648)

personal characteristics	*N*	%
**Age**		
< 20	169	26.1
20	133	20.5
> 20	346	53.4
Mean ± SD.	20.48 ± 1.62	
**Gender**		
Male	340	52.5
Female	308	47.5
**Academic grades**		
Grade 1	159	24.5
Grade 2	103	15.9
Grade 3	202	31.2
Grade 4	184	28.4
**Living in**		
Home	394	60.8
Dormitory	217	33.5
Other	37	5.7
**Receiving training on disaster**		
Yes	65	10.0
No	583	90.0
**Duration of training (n = 65)**		
One day	17	26.2
3days	23	35.4
One weak	14	21.5
More than one weak	11	16.9
**Number of courses (n = 65)**		
One	50	76.9
Two	11	16.9
More than two	4	6.2

Abbreviation: N, total number of respondents, n (%) number and percentage of nursing students to each category, data represent as frequency and percentage, SD: Standard deviation

### Knowledge and self-efficacy enhancement through educational implementation

Table 2 shows that the total mean score of knowledge significantly improved from 19.93 ± 5.66 pre-implementation phase to 34.53 ± 7.58 post-implementation phase. Also, the average knowledge mean score improved from 0.50 ± 0.14 to 0.86 ± 0.19, reinforcing the positive effect. As regards total mean score of mean self-efficacy mean scores also improved significantly, rising from 60.88 ± 15.12 to 83.27 ± 13.59. while the average self-efficacy score increased from 3.20 ± 0.80 to 4.38 ± 0.72, indicating a strong enhancement in students’ confidence levels. Moreover, improvement in satisfactory knowledge and positive self-efficacy suggests that the intervention was highly effective in enhancing students’ understanding and confidence with statistically significant differences between the program phases (*p* < 0.001).

**Table 2:** Distribution of the studied mother according to overall knowledge and Self-Efficacy (*N* = 648)

Knowledge	Pre		Post		Test of Sig.	*p*	
	No.	%	No.	%			
Unsatisfactory (≤ 50%)	296	45.7	36	5.6	MH = 1220.0^*^	< 0.001^*^	
Average (51:75%)	347	53.5	126	19.4			
Satisfactory (76: 100%)	5	0.8	486	75.0			
**Total Score (0–40)**							
Min. – Max.	5.0–32.0		8.0–40.0		t = 39.721^*^	< 0.001^*^	
Mean ± SD.	19.93 ± 5.66		34.53 ± 7.58				
**Average Score (0–1)** ** (Mean ± SD.)**	0.50 ± 0.14		0.86 ± 0.19				
**Self-Efficacy**							
Negative (< 3)	255	39.4	36	5.6	MH = 634.000^*^		< 0.001^*^
Basically Confidence (= 3)	37	5.7	3	0.5			
Positive (> 3)	356	54.9	609	94.0			
**Total Score (19–95)**							
Min. – Max.	19.0–95.0		19.0–95.0		t = 27.096^*^		< 0.001^*^
Mean ± SD.	60.88 ± 15.12		83.27 ± 13.59				
**Average Score (1–5)** ** (Mean ± SD.)**	3.20 ± 0.80		4.38 ± 0.72				

SD: Standard deviation t: Paired t-test

MH: Marginal Homogeneity Test

p: p value for comparing between pre and post

*: Statistically significant at *p* ≤ 0.05

### Impact of demographic factors on disaster-related knowledge acquisition

Significant differences were observed in Table 3 regarding living arrangements, prior disaster training, and the number/duration of training courses in the post-intervention phase. However, there were no statistically significant differences in total knowledge scores concerning age, gender, and academic level in relation to personal characteristics (*p* > 0.05).

**Table 3:** Relation between level of knowledge and personal characteristics **(N = 648)**

Knowledge												
Personal characteristics	Pre						Post					
	Unsatisfactory (*n* = 296)		Average (*n* = 347)		Satisfactory (*n* = 5)		Unsatisfactory (*n* = 36)		Average (*n* = 126)		Satisfactory (*n* = 486)	
	**No.**	**%**	**No.**	**%**	**No.**	**%**	**No.**	**%**	**No.**	**%**	**No.**	**%**
**Age**												
< 20	89	52.7	77	45.6	3	1.8	22	13.0	64	37.9	83	49.1
20	55	41.4	77	57.9	1	0.8	3	2.3	17	12.8	113	85.0
> 20	152	43.9	193	55.8	1	0.3	11	3.2	45	13.0	290	83.8
**χ** ^**2**^ **(p)**	**8.536 (** ^**MC**^ **p=0.054)**						83.429^*^(< 0.001^*^)					
**Gender**												
Male	193	56.8	145	42.6	2	0.6	21	6.2	81	23.8	238	70.0
Female	103	33.4	202	65.6	3	1.0	15	4.9	45	14.6	248	80.5
**χ** ^**2**^ **(p)**	**35.779** ^*^ **(** ^**MC**^ **p<0.001** ^*^ **)**						**9.935** ^*^ **(0.007** ^*^ **)**					
**Academic level**												
Level 1	85	53.5	72	45.3	2	1.3	22	13.8	64	40.3	73	45.9
Level 2	45	43.7	57	55.3	1	1.0	0	0.0	2	1.9	101	98.1
Level 3	87	43.1	113	55.9	2	1.0	9	4.5	41	20.3	152	75.2
Level 4	79	42.9	105	57.1	0	0.0	5	2.7	19	10.3	160	87.0
**χ** ^**2**^ **(p)**	**7.998** (^**MC**^**p=0.185)**						116.777^*^ (< 0.001^*^)					
**Where Living in**												
Home	172	43.7	218	55.3	4	1.0	26	6.6	81	20.6	287	72.8
Dormitory	103	47.5	113	52.1	1	0.5	9	4.1	37	17.1	171	78.8
Other	21	56.8	16	43.2	0	0.0	1	2.7	8	21.6	28	75.7
**χ** ^**2**^ **(p)**	**3.064 (** ^**MC**^ **p=0.537)**						**3.735 (0.443)**					
**Taking Disaster Training course Before**												
**Yes**	**33**	**50.8**	**31**	**47.7**	**1**	**1.5**	**4**	**6.2**	**12**	**18.5**	**49**	**75.4**
No	263	45.1	316	54.2	4	0.7	32	5.5	114	19.6	437	75.0
**χ** ^**2**^ **(p)**	**2.057** (^**MC**^**p=0.286)**						**0.084 (0.959)**					
**Duration of course (n = 65)**	**(n = 33)**		**(n = 31)**		**(n = 1)**		**(n = 4)**		**(n = 12)**		**(n = 49)**	
One day	6	35.3	11	64.7	0	0.0	0	0.0	1	5.9	16	94.1
3days	10	43.5	12	52.2	1	4.3	2	8.7	6	26.1	15	65.2
One weak	11	78.6	3	21.4	0	0.0	1	7.1	3	21.4	10	71.4
More than one weak	6	54.5	5	45.5	0	0.0	1	9.1	2	18.2	8	72.7
**χ** ^**2**^ **(p)**	**8.537** (^**MC**^**p=0.138)**						**5.278** (^**MC**^**p=0.478)**					
**Number of courses (n = 65)**	**(n = 33)**		**(n = 31)**		**(n = 1)**		**(n = 4)**		**(n = 12)**		**(n = 49)**	
One	26	52.0	24	48.0	0	0.0	2	4.0	8	16.0	40	80.0
Two	6	54.5	4	36.4	1	9.1	2	18.2	2	18.2	7	63.6
More than two	1	25.0	3	75.0	0	0.0	0	0.0	2	50.0	2	50.0
**χ** ^**2**^ **(p)**	**6.057** (^**MC**^**p=0.257)**						**5.864** (^**MC**^**p=0.145)**					

χ^2^: Chi square test MC: Monte Carlo

p: p value for comparison between the studied categories

*: Statistically significant at *p* ≤ 0.05

### Impact of structured disaster education on nursing students’ self-efficacy

Table 4 highlights significant differences in self-efficacy levels concerning academic level (*p* < 0.001) in both pre- and post-intervention phases and living arrangements (*p* = 0.002) in the pre-intervention phase. However, there were no statistically significant correlations discovered between self-efficacy and age, gender, prior disaster training, or the number and duration of training courses in either phase (*p* > 0.05).

**Table 4:** Relation between level of Self-Efficacy and personal characteristics **(N = 648)**

	Self-Efficacy											
Personal characteristics	Pre						Post					
	Negative (*n* = 255)		Basicly Cnfidence (*n* = 37)		Positive (*n* = 356)		Negative (*n* = 36)		Basicly Cnfidence (*n* = 3)		Positive (*n* = 609)	
	**No.**	**%**	**No.**	**%**	**No.**	**%**	**No.**	**%**	**No.**	**%**	**No.**	**%**
**Age**												
< 20	68	40.2	13	7.7	88	52.1	27	16.0	3	1.8	139	82.2
20	47	35.3	11	8.3	75	56.4	2	1.5	0	0.0	131	98.5
> 20	140	40.5	13	3.8	193	55.8	7	2.0	0	0.0	339	98.0
**χ** ^**2**^ **(p)**	**6.036 (0.196)**						**45.669** ^*^ **(** ^**MC**^ **p<0.001** ^*^ **)**					
**Gender**												
Male	124	36.5	22	6.5	194	57.1	25	7.4	1	0.3	314	92.4
Female	131	42.5	15	4.9	162	52.6	11	3.6	2	0.6	295	95.8
**χ** ^**2**^ **(p)**	**2.820 (0.244)**						**4.874 (** ^**MC**^ **p=0.053)**					
**Academic level**												
Level 1	60	37.7	13	8.2	86	54.1	27	17.0	3	1.9	129	81.1
Level 2	43	41.7	6	5.8	54	52.4	0	0.0	0	0.0	103	100.0
Level 3	50	24.8	15	7.4	137	67.8	6	3.0	0	0.0	196	97.0
Level 4	102	55.4	3	1.6	79	42.9	3	1.6	0	0.0	181	98.4
**χ** ^**2**^ **(p)**	42.459^*^(< 0.001^*^)						**50.402** ^*^ **(** ^**MC**^ **p<0.001** ^*^ **)**					
**Where Living in**												
Home	140	35.5	23	5.8	231	58.6	28	7.1	3	0.8	363	92.1
Dormitory	107	49.3	12	5.5	98	45.2	7	3.2	0	0.0	210	96.8
Other	8	21.6	2	5.4	27	73.0	1	2.7	0	0.0	36	97.3
**χ** ^**2**^ **(p)**	**16.856** ^*^ **(0.002** ^*^ **)**						**5.883 (** ^**MC**^ **p=0.175)**					
**Taking Disaster Training course Before**												
** Yes**	**24**	**36.9**	**0**	**20.0**	**41**	**63.1**	**1**	**1.5**	**0**	**0.0**	**64**	**98.5**
No	231	39.6	37	6.3	315	54.0	35	6.0	3	0.5	545	93.5
**χ** ^**2**^ **(p)**	**5.105** (**0.078)**						**2.111 (** ^**MC**^ **p=0.398)**					
**Duration of course (n = 65)**	**(n = 24)**		**(n = 0)**		**n = 41)**		**(n = 1)**		**(n = 0)**		**(n = 64)**	
One day	9	52.9	–	–	8	47.1	0	0.0	–	–	17	100.0
3days	6	26.1	–	–	17	73.9	0	0.0	–	–	23	100.0
One weak	5	35.7	–	–	9	64.3	0	0.0	–	–	14	100.0
More than one weak	4	36.4	–	–	7	63.6	1	9.1	–	–	10	90.9
**χ** ^**2**^ **(p)**	**3.043** (**0.385)**						**3.778** (^**MC**^**p=0.171)**					
**Number of courses (n = 65)**	**(n = 24)**		**(n = 0)**		**n = 41)**		**(n = 1)**		**(n = 0)**		**(n = 64)**	
One	22	44.0	0	0.0	28	56.0	0	0.0	0	0.0	50	100.0
Two	1	9.1	0	0.0	10	90.9	1	9.1	0	0.0	10	90.9
More than two	1	25.0	0	0.0	3	75.0	0	0.0	0	0.0	4	100.0
**χ2(p)**	**6.057** (^**MC**^**p=0.257)**						**4.735** (^**MC**^**p=0.226)**					

χ2: Chi square test MC: Monte Carlo

p: p value for comparison between the studied categories

*: Statistically significant at *p* ≤ 0.05

### Knowledge and Self-Efficacy correlation

Table 5 showed there was a statistically significant positive correlation between the knowledge of studied students and self-efficacy in both pre-and post-intervention. Pre-intervention, a weak but statistically significant correlation was observed (*r* = 0.137, *p* < 0.001), suggesting that higher knowledge alone did not strongly predict confidence in disaster response. However, post-intervention, the correlation strengthened considerably (*r* = 0.608, *p* < 0.001), indicating a more substantial alignment between knowledge acquisition and self-efficacy.

**Table 5:** Correlation between knowledge and self-efficacy (N = 648)

	Pre		Post	
	*r*	*p*	*r*	*p*
**Knowledge vs. self-efficacy**	0.137^*^	< 0.001^*^	0.608^*^	< 0.001^*^

r: Pearson coefficient

*: Statistically significant at *p* ≤ 0.05

## Discussion

Disaster management is a structured process that focuses on effective preparation and response to disasters by strategically managing resources to minimize harm, mitigate potential losses, ensure timely assistance to victims, and facilitate recovery [13]. Disaster nursing education plays a vital role in enhancing healthcare resilience by ensuring that nurses at all levels are adequately prepared to respond to crises [18]. This study assessed the effectiveness of the Jennings Disaster Management Model in improving nursing students’ knowledge and self-efficacy in disaster response.

The findings of this study indicate a significant improvement in nursing students’ knowledge scores regarding disaster-related concepts, classifications, management strategies, and preventive and rehabilitation measures in the post-test phase. Additionally, statistically significant differences were observed in the total knowledge and self-efficacy scores between the pre-test and post-test phases.

This study demonstrated a statistically significant increase in knowledge levels (*p* < 0.001). Before the intervention, 45.7% of students exhibited unsatisfactory knowledge (≤ 50%), while only 0.8% achieved satisfactory levels (≥ 76%). However, following the intervention, 75% of students attained satisfactory knowledge, and the proportion of those with unsatisfactory knowledge significantly declined to 5.6%. These findings align with those of Arun Jothi (2015), who conducted a study at the School of Nursing in Kanyakumari District. In the pre-test phase, 46% of students had inadequate knowledge, 44% demonstrated moderate knowledge, and only 10% possessed adequate knowledge. Post-intervention, 76% of students achieved adequate knowledge, while the remaining 24% demonstrated moderate understanding [16].

Similarly, these findings align with Hamdi and Al Thobaity (2023) who examined a structured disaster training intervention at Taif University, Saudi Arabia, revealing notable post-training improvements in disaster knowledge [19]. Consistently, Wulandari et al. (2023) found that while 57% of nursing students in Indonesia demonstrated high disaster awareness, targeted interventions remain essential to address existing knowledge deficits [20].

Furthermore, this study highlighted a substantial improvement in self-efficacy scores post-intervention, with most students shifting from limited confidence levels to strong self-efficacy. This aligns with Wong et al. (2022), who emphasized that experiential learning plays a crucial role in students’ ability to retain and apply disaster management principles in real-world scenarios. Given the increasing frequency and complexity of global disasters, it is essential to ensure that nursing students acquire adequate preparedness skills [21].

Further supporting these findings by Park and Hwang (2024) conducted quasi-experimental study to evaluate the effectiveness of simulation-based education is further corroborated whose demonstrated statistically significant gains (*p* < 0.001) in disaster nursing competencies, triage accuracy, critical thinking, and preparedness [22]. This reinforces the imperative need to integrate high-fidelity simulations into nursing curricula, bridging the gap between theoretical knowledge and practical response capabilities. Supporting this, Koca and Arkan (2020) employed the Jennings Model alongside a Learning Management System, yielding a significant increase in nursing students’ disaster preparedness perceptions and self-efficacy (*p* < 0.05) [23].

The impact of structured disaster education is further validated by Ma et al. (2021) whose randomized controlled trial in China demonstrated statistically significant improvements across cognitive, skill-based, and affective domains of disaster competence (*p* < 0.01) [24].

The consistency of these findings extends to Mirzaei et al. (2020), whose study at Shahid Rahnemoon Hospital, Iran, showed a statistically significant rise in nurses’ disaster readiness (*p* < 0.001) [25], and Xia et al. (2020), who reported notable improvements in disaster knowledge among hospital-based nursing trainees in China (*p* = 0.003) [26]. Additionally, Davis et al. (2020) highlighted the transformative impact of high-fidelity simulation scenarios, demonstrating substantial post-training knowledge gains among nursing students in the United States [27].

Further reinforcing this study’s outcomes, Dastyar et al. (2023) demonstrated that disaster training significantly improved self-efficacy scores among Iranian nursing students (*p* < 0.001) [28], while Kılıç and Şimşek (2019) found a notable increase in general self-efficacy post-training (*p* < 0.01) [29]. Likewise, Hasan et al. (2024) reported high self-efficacy levels among Bangladeshi nurses despite moderate disaster knowledge, indicating the need for holistic training that strengthens both confidence and practical competencies [30].

Contrary to our findings, Çiriş Yildiz and Yildirim (2024) reported no significant differences in disaster response self-efficacy scores following an educational intervention, suggesting that curriculum design, instructional methods, and duration may influence training efficacy [31].

Similarly, no statistically significant associations were found between self-efficacy and demographic variables such as age, gender, prior disaster training, or the number and duration of training courses. These results mirror the findings of Kim and Lee (2023), who reported that well-structured disaster education programs enhance self-efficacy regardless of demographic characteristics [32].

In contrast, Soylu et al. (2025), reported that age, gender, educational level, and disaster training as significant determinants of response self-efficacy (*P* < 0.05) [33]. However, our study found no statistically significant associations between self-efficacy and these variables (*p* >0.05), with the exception of academic level, which showed a highly significant impact (*P* < 0.001). This challenges the widely held belief that demographic characteristics and prior disaster training alone can enhance response confidence. These conflicting results underscore the urgent need for deeper investigation into alternative factors that shape self-efficacy in disaster preparedness, paving the way for more targeted and evidence-based educational interventions.

Moreover, Wang et al. (2022) highlighted that experiential learning and simulation-based training play a more pivotal role in fostering disaster response confidence than demographic factors alone. These findings reinforce the need to integrate structured disaster education within nursing curricula to strengthen self-efficacy across diverse student populations [21].

Finally, this study reinforces the critical need to integrate disaster nursing courses into nursing education. The findings align with Erkin and Kiyan (2025), who evaluated the impact of disaster nursing education on students’ disaster literacy and preparedness perceptions. Their research demonstrated that embedding disaster nursing into the curriculum led to a profound enhancement in students’ disaster literacy and preparedness [34].

In terms of feasibility, the training program’s comprehensive content makes it well-suited for integration in the third academic year, where students possess a solid foundation and clinical context. Alternatively, the training can be divided and incorporated progressively throughout the four academic years. Implementation would require a team of trained faculty members to deliver the content effectively, which aligns with long-term curriculum development strategies in nursing education.

Variations in self-efficacy could be attributed to differences in prior knowledge, clinical exposure, and confidence in managing emergencies. Future research should explore the long-term effects of disaster education and examine additional factors—such as personality traits and coping mechanisms—that may contribute to differences in self-efficacy development.

### Study limitations

One notable limitation is that data collection relied on an online questionnaire, which may have introduced response errors due to internet-based biases. Furthermore, the use of convenience sampling from a single institution restricts the generalizability of the findings. Since the data were solely self-reported and based on specific research scales, the possibility of response bias cannot be overlooked.

Additionally, conducting the study within a single state university limits its applicability to nursing students nationwide. More importantly, the absence of a control group hinders direct comparisons between students who completed the disaster nursing course and those who did not. To enhance reliability and applicability, future studies should incorporate a control group and a more diverse sample across multiple institutions.

## Conclusion

This study emphasizes the effectiveness of structured disaster preparedness education in significantly enhancing nursing students’ knowledge and self-efficacy. The findings revealed a notable improvement in knowledge scores post-intervention, with self-efficacy levels also showing significant growth. These results affirm the positive impact of integrating targeted disaster education within nursing curricula.

Demographic factors, such as prior disaster training and living arrangements, influenced knowledge acquisition, whereas variables like age, gender, and academic level showed no significant association. Additionally, a strong positive correlation between knowledge and self-efficacy highlights the importance of both theoretical learning and confidence-building measures in disaster preparedness.

Given these findings, future research should explore long-term knowledge retention, assess real-world application of disaster response skills, and incorporate advanced training methods, such as virtual reality and AI-driven simulations. Standardizing disaster education frameworks across nursing programs will further ensure that future nurses are equipped to respond effectively and confidently in high-stakes disaster scenarios.

## Supplementary Information

Below is the link to the electronic supplementary material.

Supplementary Material 1

Supplementary Material 2

## Data Availability

The datasets generated and analyzed during this study are available from the corresponding author upon reasonable request.

## Abbreviations

JDMM: Jenning Disaster Management Model
DMKQ: Disaster Management Knowledge Questionnaire
DRSES: Disaster Response Self-Efficacy Scale
ICN: International Council of Nurses
MCQs: Multiple-Choice Questions
WHO: World Health Organization
CDC: Centers for Disease Control and Prevention
SPSS: Statistical Package for the Social Sciences
COPE: Committee on Publication Ethics
