# Supplementary material for: Evaluating the effectiveness of the Jennings disaster management model on nursing students’ knowledge and self-efficacy
Source: BMC Nurs. 2025 Dec 11;24:1476. doi: 10.1186/s12912-025-04098-2 (PMC12696946; doi:10.1186/s12912-025-04098-2)
Supplement: Supplementary file 1 — Supplementary Material 1 [file 12912_2025_4098_MOESM1_ESM.docx]

**DISASTER MANAGEMENT KNOWLEDGE QUESIONAIR**

***INSTRUCTION***

Read the following items carefully and select one correct response by

placing the appropriate tick mark in the space provided.

***Section 1 : Demographic characteristics***

- Age

( years)

- Gender
- Female ( ) Male ( )
- Academic level
- level 1 ( ) level 2 ( ) level 3 ( ) level 4( )
- Where Living in
- Home ( )
- Dormitory ( )
- Other ( )
- Taking Disaster Training course Before
- Yes( )
- No ( )
- Duration of course
- One day ( )
- 3days( )
- One weak( )
- More than one weak ( )
- Number of courses ( )

**Section 2: Knowledge regarding concept of disaster**

**1. What is a disaster?**

a) Changing event

b) Situational event

c) Calamitous event

d) Traumatic event

**2. What are all the features of disaster?**

i. . The surprise is in the timing

ii. The lack of time available to take the necessary decisions to confront them

iii. Threatening the higher national interests.

a) (i), (ii)

b) (i), (iii)

c) (ii), (iii)

d) (i), (ii), (iii)

**3. How many phases are there in disaster?**

a) Three

b) Four

c) Five

d) Six

**4. The number of injuries and death during disaster deepened on?**

a) Types, density, distribution, and degree of the preparedness.

b) Types, density.

c) Density, distribution

d) None of the above

**5. What are the effect of disaster?**

a) Premature deaths.

b) Environmental imbalance

c) Nutritional deficiencies.

d) All of the above.

**6. What is the common disease found after flood?**

a) malaria.

b) Diarrhea and diseases of the gastrointestinal tract.

C) measles.

d) All of the above.

**7. What are the health effects of massive chemical exposure?**

a) Disease cluster and deaths.

b) Air pollution.

c) Contamination of food and water

d) None of the above

**8. What are all the effects of biological disaster?**

a) New unknown disease

b) Known disease

c) Chronic disease

d) All of the above

**9 .Which ministry is responsible for air accident?**

a) Ministry of health

b) Ministry of home affairs

c) Ministry of civil aviation

d) Ministry of environment and forests.

**10. The type of disaster more prevalent in Egypt is ……………………….**

a) Flood

b) Drought

c) Landslides

d) Aviation

**11.Which are the international agencies providing health humanitarian assistance?**

a) WHO

b) FAO

c) ECHO

d) All of the above

***Section 3: Knowledge regarding types of disaster.***

**12.What are all examples of natural disaster?**

a) Riots, tornado, earth quake

b) Terrorism, bombing, fire

c) Expulsion, intended damage to properties, riots.

d) Tornado, volcanic eruption, earth quake.

**13.What among the following is not considered as a slow Disaster?**

a) Drought

b) Food shortage

c) Famine

d) Landslide

**14.What are the examples of manmade disaster?**

a) Bombing, plane crashing

b) Landslide, tempest

c) Tornado, twister

d) Epidemics, agricultural pests

***Section 4: Knowledge regarding disaster management***

15.What is disaster management?

a) Development of recovery plans

b) Develop the recovery plan and implementation

c) Implementation of plan

d) Prevention of disaster.

**16.What one of the following is not a principle of disaster management?**

a) Prevent the occurrence

b) Minimize the number of causalities

c) Rescue the victim

d) Identify the person and area.

**17.What dose disaster preparedness refers to?**

a) Action taken to prevent or reduce the harmful effect of disaster.

b) Activities to prevent subsequent disaster

c) Involves plans of being ready, properly equipped to face disaster

d) Being prepared to reduce the future disaster.

**18.Among the following what is not correct response during pre-impact period?**

a) Fore cast

b) Early warning

c) Preparedness

d) Monitoring of impact

**19.Following are the disaster management in impact phase EXCEPT.**

a) Medical care

b) Supply of food and water

c) Supply of essential items

d) Estimating loss of life and property

**20.Name the process of classifying the sick and the injured according to the urgency and type of condition?**

a) Damage assessment

b) Emergency assessment

c) Triaging process

d) Vulnerability assessment

**21.How many categories are there in triage?**

a) Two

b) Three

c) Four

d) Five

**22.What condition will not come under high priority?**

a) Respiratory arrest

b) Conscious patient with head injury

c) Open chest wound

d) Burns involving respiratory tract

**23.What are the conditions comes under lowest priority?**

a) Minor bleeding and wound

b) Obvious death

c) Moderate burns

d) All of the above

**24.What is the significance of red tag in triaging process?**

a) Fire

b) Activate the emergency plan

c) Evacuation

d) The bomb threat

**25.If yellow tag is used in color cording, what does it imply on treatment priority?**

a) Require immediate treatment

b) Second priority

c) Third priority

d) Fourth priority

**26.How the bomb threat disaster be announced?**

a) Code yellow

b) Code brown

c) Code grey

d) Code black

**27.Who are the members in disaster team?**

a) Administrative officers, police

b) Physicians, nurses, rescue team

c) Volunteers

d) All of the above

**28.When triage starts or begins?**

a) Disaster site

b) Patient enters in hospital

c) During transportation

d) Preparedness

**29.What is not a suitable measure to manage the toxic chemical?**

a) Identification of the hazard

b) Assessment of the risk to the community

c) Safe measures and alternatives

d) Health education

**30.What is the disaster management in flood?**

a) Turn off the electricity

b) Protect people

c) Beware of water contamination

d) All of the above

**31.What are measures that should be taken during cloud of toxic fumes?**

a) Close the doors and windows

b) Stop up air intake

c) Turn off ventilation and air conditioners

d) All of the above

**32.What measures to be following for personal protection?**

a) Do not use the telephone except to call

b) Listen to the messages broad cast

c) Keep an emergency kit ready

d) All of the above

**33.What is not be done in case of earthquake?**

a) Go back to the damage area

b) Avoid storing heavy object and materials in height

c) Stay away from the stairs

d) Prepare a family emergency kit

***Section 5: Knowledge regarding preventive measures and rehabilitation***

***measures in disaster***

**34.What are the disaster prevent measures?**

a) Prevent human settlement in low-lying area

b) Improvement of warning system

c) Hazard-proof roads, bridges, canal, water reservoirs, power transmission lines.

d) All of the above

**35.Among the following, which is not a relevant, hygienic and sanitation measures after a disaster?**

a) Immunization

b) Disposal of waste and excreta

c) Fly proofing and rodents control.

d) Health education.

**36.How much dosage of chlorine tablet is used for 1 litter of drinking water?**

a) 2.5 gm.

b) 2 gm.

c) 0.5 mg.

d) 0.125 mg.

**37.In which type of disaster the immunization will be indicated?**

a) Biological disasters

b) Chemical disasters

c) Drought

d) All of the above

**38.Among the following, which is not a relevant to vaccination?**

i) Active immune

ii) Passive immune

iii) Cold-chain

iv) Develop resistant

a) (i) (ii) (iii) Active immune_Passive immune_ Coldchain

b) (ii) (iii) (iv) Passiveimmune_Cold-chain_Develop resistant

c) (i) (iii) (iv) Activeimmune_Cold-chain_Develop resistant

d) (i) (ii) (iv) i) Active immune_Passive immune_Develop resistant

**39.What does disaster rehabilitation refer to?**

a) Restore the pre occurrence of event

b) Restore the occurrence of event

c) Prevent the occurrence of events

d) Promote the occurrence of events

**40.What is the disaster management in rehabilitation phase?**

a) Temporary shelter and supply of water, food, cloths.

b) Repair of roads, electricity, and communication net work.

c) Restoration of health and educational facilities.

d) All of the above.
