# Supplementary material for: Evaluating the effectiveness of the Jennings disaster management model on nursing students’ knowledge and self-efficacy
Source: BMC Nurs. 2025 Dec 11;24:1476. doi: 10.1186/s12912-025-04098-2 (PMC12696946; doi:10.1186/s12912-025-04098-2)
Supplement: Supplementary file 2 — Supplementary Material 2 [file 12912_2025_4098_MOESM2_ESM.docx]

**Disaster Response Self-Efficacy Scale**

Instructions: This scale measures your disaster response self-efficacy or how confident you are in

your ability to respond during disasters. Please rate the following items based on the level of

your confidence and competence in doing them.

| **NO.** | **Items** | **(1)**  **Have no**  **confidence**  **at all** | **(2)**  **Basically**  **have no**  **confidence** | **(3)**  **Have a little**  **confidence** | **(4)**  **Basically**  **have**  **confidence** | **‎(5)‎**  **Have**  **complete**  **confidence** |
| --- | --- | --- | --- | --- | --- | --- |
| 1 | Assess the relative harm from the disaster |  |  |  |  |  |
| 2 | Assess injuries accurately and swiftly |  |  |  |  |  |
| 3 | Assess the epidemic situation after the disaster, such as infectious diseases or acute poisoning |  |  |  |  |  |
| 4 | Recognize vulnerable groups, such as chronic patients or disabled people |  |  |  |  |  |
| 5 | Triage technique |  |  |  |  |  |
| 6 | Debridement, hemostasis, bandaging, and splinting |  |  |  |  |  |
| 7 | Lifting |  |  |  |  |  |
| 8 | Transfer |  |  |  |  |  |
| 9 | Emergency rescue techniques (BLS) |  |  |  |  |  |
| 10 | Intensive care and nursing of critically ill patients |  |  |  |  |  |
| 11 | Prevention and control of infectious diseases in disaster areas |  |  |  |  |  |
| 12 | Initial psychological assessment of disaster victims |  |  |  |  |  |
| 13 | Recognize common psychiatric and psychological problems after disaster, such as PTSD, depression, and anxiety |  |  |  |  |  |
| 14 | Provide basic Psychological treatment for disaster victims |  |  |  |  |  |
| 15 | Referral of victims who need psychiatric and psychological treatment in the disaster area |  |  |  |  |  |
| 16 | Adjust psychological state of self and adapt to the working environment quickly |  |  |  |  |  |
| 17 | Communicate with other team  professionals and establish good cooperation relationship |  |  |  |  |  |
| 18 | Actively communicate with victims and relatives and establish a good nurse-patient relationship |  |  |  |  |  |
| 19 | Obey professional ethics, remain humanitarian and full of empathy and love |  |  |  |  |  |
